# Cardio-Respiratory synchronized bSSFP MRI for high throughput in vivo lung tumour quantification

**DOI:** 10.1371/journal.pone.0212172

**Published:** 2019-02-12

**Authors:** Ana L. Gomes, Paul Kinchesh, Stuart Gilchrist, Philip D. Allen, Luiza Madia Lourenço, Anderson J. Ryan, Sean C. Smart

**Affiliations:** Cancer Research UK and Medical Research Council Oxford Institute for Radiation Oncology, Department of Oncology, University of Oxford, Oxford, United Kingdom; Northwestern University Feinberg School of Medicine, UNITED STATES

## Abstract

The identification and measurement of tumours is a key requirement in the study of tumour development in mouse models of human cancer. Disease burden in autochthonous tumours, such as those arising in the lung, can be seen with non-invasive imaging, but cannot be accurately measured using standard tools such as callipers. Lung imaging is further complicated in the mouse due to instabilities arising from the rapid but cyclic cardio-respiratory motions, and the desire to use free-breathing animals. Female A/JOlaHsd mice were either injected (i.p.) with PBS 0.1ml/10g body weight (n = 6), or 10% urethane/PBS 0.1ml/10g body weight (n = 12) to induce autochthonous lung tumours. Cardio-respiratory synchronised bSSFP MRI, at 200 μm isotropic resolution was performed at 8, 13 and 18 weeks post induction. Images from the same mouse at different time points were aligned using threshold-based segmented masks of the lungs (ITK-SNAP and MATLAB) and tumour volumes were determined via threshold-based segmentation (ITK-SNAP).Scan times were routinely below 10 minutes and tumours were readily identifiable. Image registration allowed serial measurement of tumour volumes as small as 0.056 mm^3^. Repetitive imaging did not lead to mouse welfare issues. We have developed a motion desensitised scan that enables high sensitivity MRI to be performed with high throughput capability of greater than 4 mice/hour. Image segmentation and registration allows serial measurement of individual, small tumours. This allows fast and highly efficient volumetric lung tumour monitoring in cohorts of 30 mice per imaging time point. As a result, adaptive trial study designs can be achieved, optimizing experimental and welfare outcomes.

## Introduction

Lung cancer is currently the major cause of cancer related deaths worldwide. Despite treatment advances in other types of cancer, current chemotherapy strategies have proven to be only marginally effective with only 10% of UK lung cancer patients surviving 5 years after diagnosis [1]. This is mainly due to patients presenting with late stage disease. Therefore, both better therapies and early diagnostic tools are needed to improve the prognosis of lung cancer patients.

Murine models of lung cancer mimicking different aspects of the human disease are already available [2], but disease progression monitoring and scoring of real-time response to therapy have proven to be more challenging. Preclinical in vivo imaging of the lung is usually done using either x-ray Computerized Tomography (CT) or Magnetic Resonance Imaging (MRI). MRI provides good spatial resolution and detection of soft tissue pathology whilst avoiding the delivery of ionising radiation as required for CT. Previous reports have suggested that lung tumours are hard to detect on CT in mice, but MRI provides good tumour to lung parenchyma contrast [3]. The requirement for CR-synchronization has also been demonstrated but in these scans there were significant periods in which data were not being acquired, compromising image capture efficiency. Regardless of the imaging speed, good correlations of tumour size by MRI with ex vivo tumour mass or diameter were given thereby validating the use of MRI in tumour volumetry for a range of scans mode, including echo-planar, FLASH, and spin-echo MRI [3–5].

MRI of the lung is challenging. The low proton density and the magnetic susceptibility induced signal losses [6], resulting from the large amount of air-tissue interface as required for gas exchange, often render the lung parenchyma invisible. However, solid tumours have relatively high proton density and do not experience these magnetic susceptibility-induced signal losses to the same extent as parenchyma. Furthermore, the effects of susceptibility can be minimised through the use of spin echo techniques of which the bSSFP scan we describe is one [7]. Another major challenge for these tumours is that the lung moves under the influence of both cardiac and respiratory motions; it is, after all functionally positioned in-between the right ventricle and the left atrium of the heart. Several 1H-based imaging methods have been developed such as hyperoxia enhancement [8, 9], inversion recovery imaging [10–12], and ultrashort [9, 13] or zero echo time (TE) [14]. Other non 1H-based methods have also been developed such as hyperpolarized helium-3 (^3^He) or xenon-129 (^129^Xe) imaging of the lung parenchyma [15–17], but these methods are expensive and some adverse effects with^129^Xe, though transient, have been reported for some patients [18]. Further complications in lung imaging are derived from the respiratory and cardiac motions, and these result in unacceptable image corruptions which may hinder or prevent the detection of pathology. The detection of small tumours in the mouse lung requires fast and high-resolution scans that are desensitised to these cardiac and respiratory motions. For the reduction of motion-derived image artefacts, retrospective gating methods have been used [19, 20] but these are necessarily slow due to the repeated acquisition of equivalent imaging data lines over multiple physiological cycles. We have previously shown that prospective gating control can be used to accelerate the production of high-resolution bSSFP (balanced Steady-State Free Precession) images of the mouse lung in vivo [21], and we now show that the technique is sensitive to the detection of small lung tumours, enabling in vivo quantification of tumour growth curves. By optimizing the gating control system, we have been able to develop a suitable Cardio-Respiratory (CR)-synchronized bSSFP scan operating at an isotropic resolution of ca. 200 μm, with an average turnaround time of <15 mins per mouse.

We demonstrate the new CR-synchronized bSSFP scan in urethane-induced model of lung cancer in the mouse. We chose this model because distinct tumours arise stochastically throughout the lung and then grow relatively slowly over a period of months. As it is a stochastic model, it is unknown when and where tumours will first be detectable. Also, as it is a slow growing model, we can test if the developed CR-synchronized bSSFP scan can detect small changes in tumour growth in vivo. The latter is likely to be important when monitoring tumour response to therapy.

## Methods

### Animal model

Experiment was conducted in accordance with the Animals Scientific Procedures Act of 1986 (UK) (Project License Number 30/3395 issued by the Home Office). The protocol was approved by the Committee on the Ethics of Animal Experiments of the University of Oxford. Mice were housed (n = 6 per cage) in individual ventilated cages in a separate room with 12h dark and light cycle maintained at 22°C in 50% humidity. Mice were given certified rodent diet, filtered water ad libitum, autoclaved bedding material and cage enrichment. No animal was euthanized on welfare grounds.

Urethane (10% urethane/PBS, 0.1mL/10g body weight, n = 12) or saline alone (0.1mL/10g body weight, n = 6) was injected i.p. in 6–8 week old A/JOlaHsd female mice weighing 20.2 ± 1.8 g (Envigo UK, #049). Urethane treated mice will henceforth be referred to as urethane mice and saline treated mice will henceforth be referred to as controls. This strain was chosen because it is described as particularly sensitive to urethane-induced lung tumours [22–24].

Six mice were killed by schedule 1 method at 24 weeks, and lungs were collected and placed in 10% buffered formalin at 4°C, for 48 h. Lungs were then transferred to 70% ethanol solution and visually inspected and surface tumours counted.

### MR imaging

MR imaging was performed at weeks 8, 13, and 18 after tumour induction. All mice were placed in a head-first supine position for imaging. Anaesthesia was induced and maintained using isoflurane (1–4%) in room air supplemented with oxygen (80%/20%). Temperature was monitored with an optical rectal probe (OTP-M, Opsens Inc, Quebec, Canada) connected via a fibre-optic extension to an AccuSense Signal Conditioner (ACS-P4-N-62SC, Opsens Inc) that provided feedback to a twisted pair resistive heating system specifically developed for MR compatible homoeothermic maintenance [25]. Respiration was monitored for prospective gating using a pressure balloon positioned against the animal’s chest. ECG was detected using subcutaneously implanted needles positioned in the lower abdomen. Throughout imaging, mice were maintained at 37°C, respiration rate was monitored and maintained at 40–60 breaths/mins with approximately 60–70% of the respiration cycle available for imaging, and all efforts were made to minimize suffering.

### MR methods

**Fig 1:**
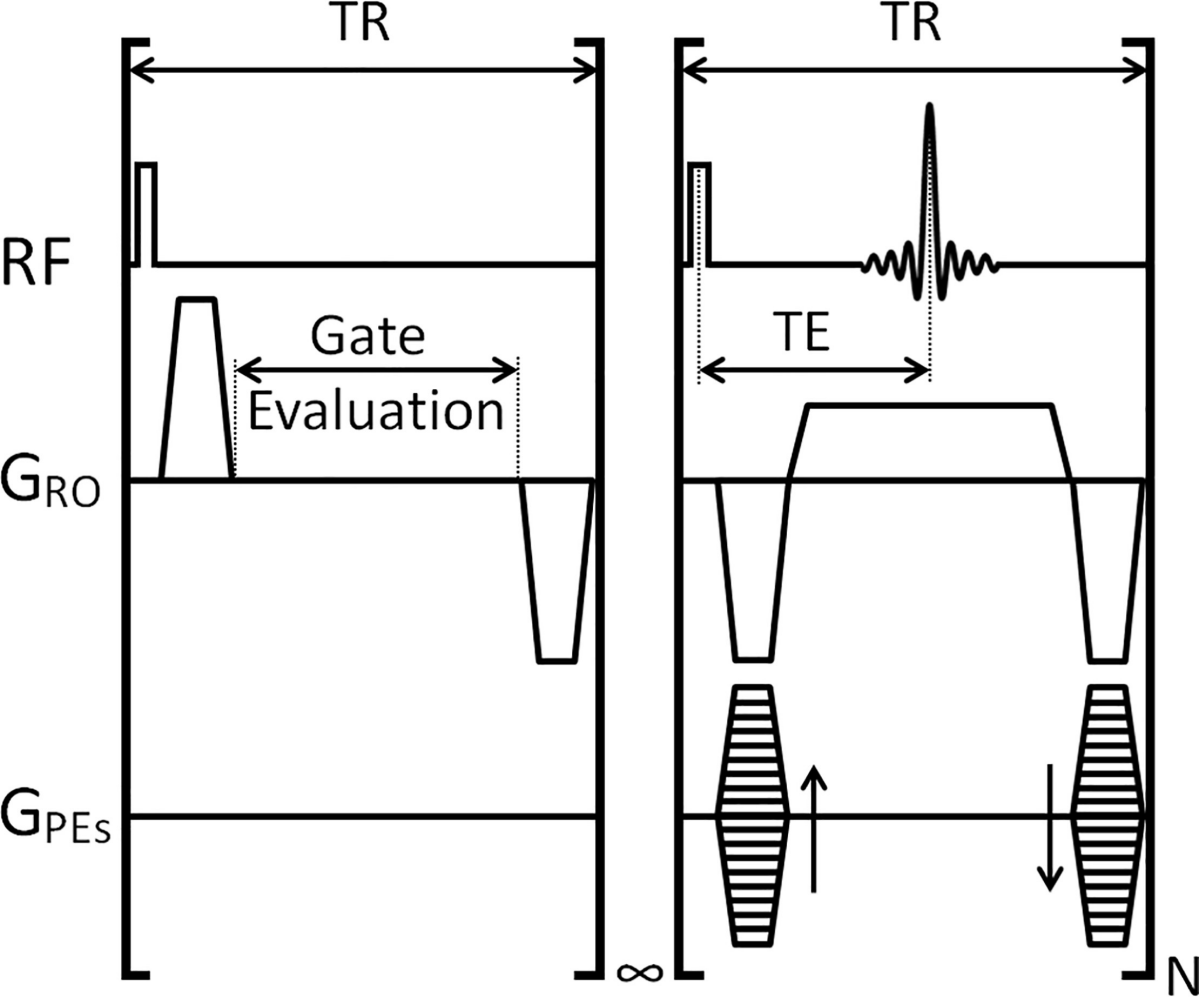
Pulse sequence schematic for the CR-synchronised bSSFP scan. The scan operates with 2 sections; in the first section the scan delivers RF and gradient pulses whilst evaluating the state of a 0 or 5-volt control signal. Upon receipt of the 5 V control signal, which is generated only from interbreath R-waves, the infinite loop is terminated and the second section, a block of N k space lines (32 in this case) were acquired before the scan reverted to the first section. Throughout the scan the steady state magnetisation was kept constant through the use of constant TR. Where a breath was identified, by a counter exceeding a time period equal to ca. 250 ms, longer than 2 heartbeats but shorter than a breath motion, then data from the 2 r-waves preceding the breath were reacquired immediately after the end of the same breath. This modus operando gives a very good desensitisation to both cardiac and respiratory motions. TR: repetition time; TE: echo time; RF: radiofrequency pulse and acquire channels; G_RO_: Readout gradient; G_PEs_: Phase-encoding gradients; N: number of phase-encoding lines per R-wave.

MRI was performed at 7.0 T (Varian VNMRS), using a 30 mm long 25 mm ID quadrature birdcage coil (Rapid Biomedical). CR-synchronised bSSFP scans were performed volumetrically with automatic and immediate reacquisition of data corrupted by respiration motion [21] and operated such that the scan automatically adapted to any instantaneous changes in respiration rate. Scan parameters were TR 2.8 ms, TE 1.4 ms, RF hard pulse 16 μs, FA 30°, FOV 51.2×25.6×25.6 mm^3^, matrix 256×128×128, and N = 32 k-lines per R-wave to give a single 3D data set with 200 μm isotropic resolution in less than 2.5 minutes. A pulse sequence schematic is given in Fig 1. bSSFP banding artefacts were robustly removed with combination of four phase-cycled images acquired in less than 10 minutes in total using an elliptical signal model [26]. All acquired scans are available at the Oxford University Research Archive Depositary (DOI: 10.5287/bodleiain:xqabzAD9e).

### Image analysis

#### Volume segmentations and image registrations

3D masks of both tumours and lungs were created by threshold-based, seed point driven, semi-automatic segmentation using ITK-SNAP [27]. Lung and tumour volume were measured from these masks. Images from the same animal at different time points were registered using an iterative closest point algorithm to find the best alignment between the corresponding lung masks [28]. Quality control of the registrations was done by quantifying the Dice Coefficients between the registered images [29]. Lung masks from the acquired scans plus the registered images/scans are available at the Oxford University Research Archive Depositary (DOI: 10.5287/bodleiain:xqabzAD9e).

### Statistical analysis

All statistical analysis was done using Excel (Office 2013). Results are shown as average and standard deviation of population. Statistical significance was assessed using two-tailed, unequal variance Student’s t-Test. P-values of less than 0.05 were considered significant.

## Results and discussion

### bSSFP MR imaging allows high throughput screening

MR bSSFP scan times, for an isotropic resolution of 200 μm, were all below 10 minutes per mouse. Including shimming, frequency offset and RF pulse calibrations the turnaround time per mouse was under 15 minutes, enabling a throughput exceeding 4 mice per hour. Such high throughput means that it would be feasible to do studies for which in vivo imaging of larger sample sizes is needed (>30 mice scanned per normal working day).

### bSSFP MR imaging allows rapid tumour burden quantification

**Fig 2:**
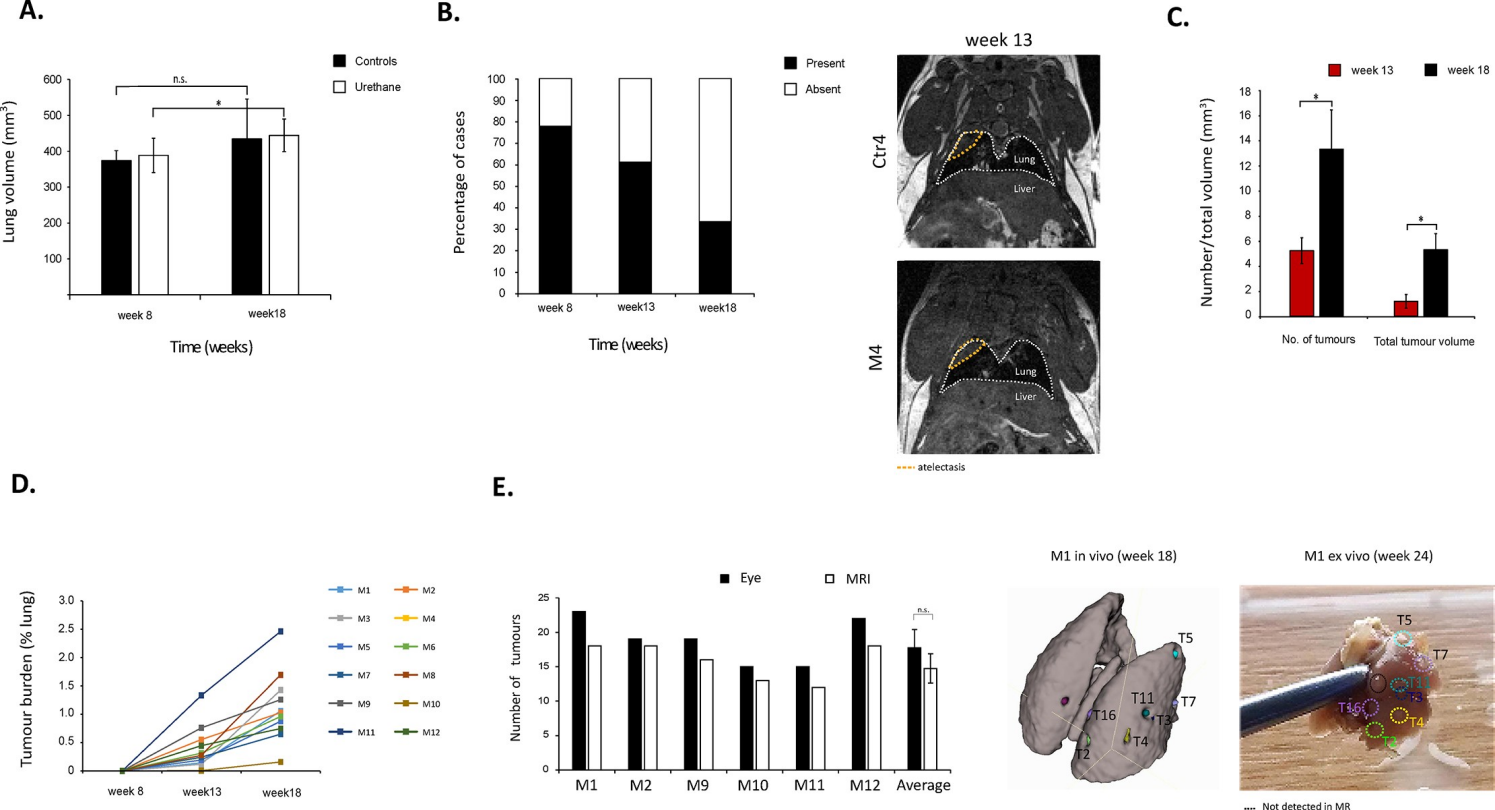
**A)** Quantification of average lung volume in mm^3^ per mouse group (control and urethane) at week 8 and 18 post induction. Error bars represent standard deviation of population. n.s.: not significant; *: ρ = 0.01. **B)** Percentage of mice with abnormal signal in the superior and/or middle right lung per time point. Right panel shows two examples of the abnormal signal (orange dotted line) observed at week 13 for mouse Control 4 (Ctr4) and urethane mouse 4 (M4). White dotted line depicts the lungs. **C)** Quantification of average of number of tumours and total tumour volume (in mm^3^), at week 13 and 18 post-induction. Error bars represent standard deviation of population. *: ρ<0.05. **D)** Quantification of tumour burden per mouse (M1-M12) at week 8, 13, and 18 post-induction. Tumour burden was defined as percentage of tumour volume per total lung volume. **E)** Comparison of quantification of number of tumours done by eye (visual inspection of surface tumours ex vivo at week 24) and MR (in vivo imaging at week 18) for individual mice (M1, M2, M9, M10, M11, and M12). Average tumour numbers obtained by both methods is also presented. Error bars represent standard deviation of population. n.s.: not significant. Right panel shows in vivo to ex vivo tumour by tumour matching for mouse M1. Black dotted line depicts a tumour observed ex vivo that had not been unequivocally identified in the in vivo MR scans.

Average lung volume for the urethane mice significantly increased from week 8 (388.55 mm^3^ ± 48.01) to week 18 (444.31 mm^3^ ± 45.34; ρ = 0.01). In control mice this difference was not significant (week 8: 373.50 mm^3^ ± 28.08; week 18: 434.18 mm^3^ ± 111.311; ρ = 0.284) (Fig 2A). The non-significance in the volume of the growing lungs in the control group is most likely attributed to the greater inter-mouse lung volume variation and the smaller number of animals in this groups, when compared to the urethane-treated group. Of note, there is partial lung volume loss in the superior and/or middle right lobes in 14 mice at week 8 (14/18, 77.8%), 11 mice at week 13 (11/18, 61.1%), and 6 mice at week 18 (6/18; 33.3%) post treatment (i.p. urethane or saline) (Fig 2B, orange dotted line on the MR scans). This partial lung volume loss was present in both treatment groups, and therefore it can be ruled out that it is part of the underlying urethane-induced pathology. Lung fibrosis can also be excluded, as the signal detected in the superior and/or middle right lobes disappears at later time points in all but two mice (Ctr4 and M5). The most likely scenario is anaesthesia induced atelectasis [30]

No tumours were unequivocally detected at week 8 post induction. The average number of tumours per urethane mouse was 5.3 ± 1.0 and 13.3 ± 2.3 at weeks 13 and 18, respectively (Fig 2C). These values are in line with previous studies published for this model [22, 31]. Also, total tumour volume was 1.2 ± 0.5 mm^3^ and 5.3 ± 1.8 mm^3^ at weeks 13 and 18, respectively (Fig 2C). All tumours measured increased in size from week 13 to week 18. No tumours were detected in any of the control mice. Quantification of individual mice tumour burden (defined as the total volume of tumours divided by the total lung volume) showed that tumours grew at different rates, in spite being in the same mouse lung (Fig 2D). For 6 urethane mice, lungs were further inspected visually post-mortem, and number of lung surface tumours per mouse were quantified by eye. Although the mean number of tumours per mouse lung was higher when quantified by visual inspection of the excised lungs (17.8 ± 2.6) at 24 weeks than by our MRI method (14.8 ± 2.1) at 18 weeks, there was no significant difference between the results obtained by both methods (ρ = 0.122; Fig 2E). Imaging was not performed at week 24 due to operational issues within the imaging facility, as a result of which we could not perform a systematic comparison of tumour number by MR with post-mortem examination at this time. It is quite conceivable that the mean number of tumours measured visually at 24 weeks is greater than the number of tumour measured by MRI at 18 weeks. This could be attributed to tumours at 18 weeks being below the limit of detection for MRI, where this limit would be primarily defined by the imaging resolution, the cell density, and the cell water content. Furthermore, whilst previous reports have indicated a good correlation of tumour volume by MRI and ex vivo mass, these have not specified any limit of detection [32, 33].

### Image registration allows individual in vivo tumour growth curves quantification

For each mouse, images measured at week 13 were registered to those measured at week 18 using a rigid body registration that was chosen as it retained volumetric detail. Images from week 8 were not registered as they did not feature tumours and as growth and/or reshaping of the lungs prevented use of the rigid body registration used. The resulting alignment improved visual identification of individual tumours and their first appearance and growth, as matched slices could be displayed simultaneously, but it did not influence measurement of tumour volumes.

The quality control of registration was assessed by quantifying Dice coefficients, as summarised in Table 1.

**Table 1:** Registration quality control check per mouse as Dice coefficients, between week 13 and week 18 scans. Numbers in bold represent successful registrations which have Dice coefficients above the pre-set registration quality control threshold.

Mouse id	Mask volume (mm^3^)		Dice coefficient
	week 13	week 18	
Ctr1	325.2	387.0	**0.786**
Ctr2	398.5	558.2	0.740
Ctr3	436.1	457.3	**0.830**
Ctr4	360.1	341.0	**0.781**
Ctr5	477.8	584.9	**0.785**
Ctr6	304.5	276.7	0.500
M1	508.0	488.3	**0.903**
M2	391.5	544.6	**0.778**
M3	468.8	416.9	**0.867**
M4	353.0	423.5	**0.784**
M5	336.4	353.1	**0.836**
M6	420.8	436.7	**0.819**
M7	433.8	452.1	**0.806**
M8	406.1	421.6	**0.809**
M9	353.2	454.7	**0.782**
M10	424.7	451.7	0.737
M11	250.0	409.8	0.560
M12	456.2	478.7	0.660

**Fig 3:**
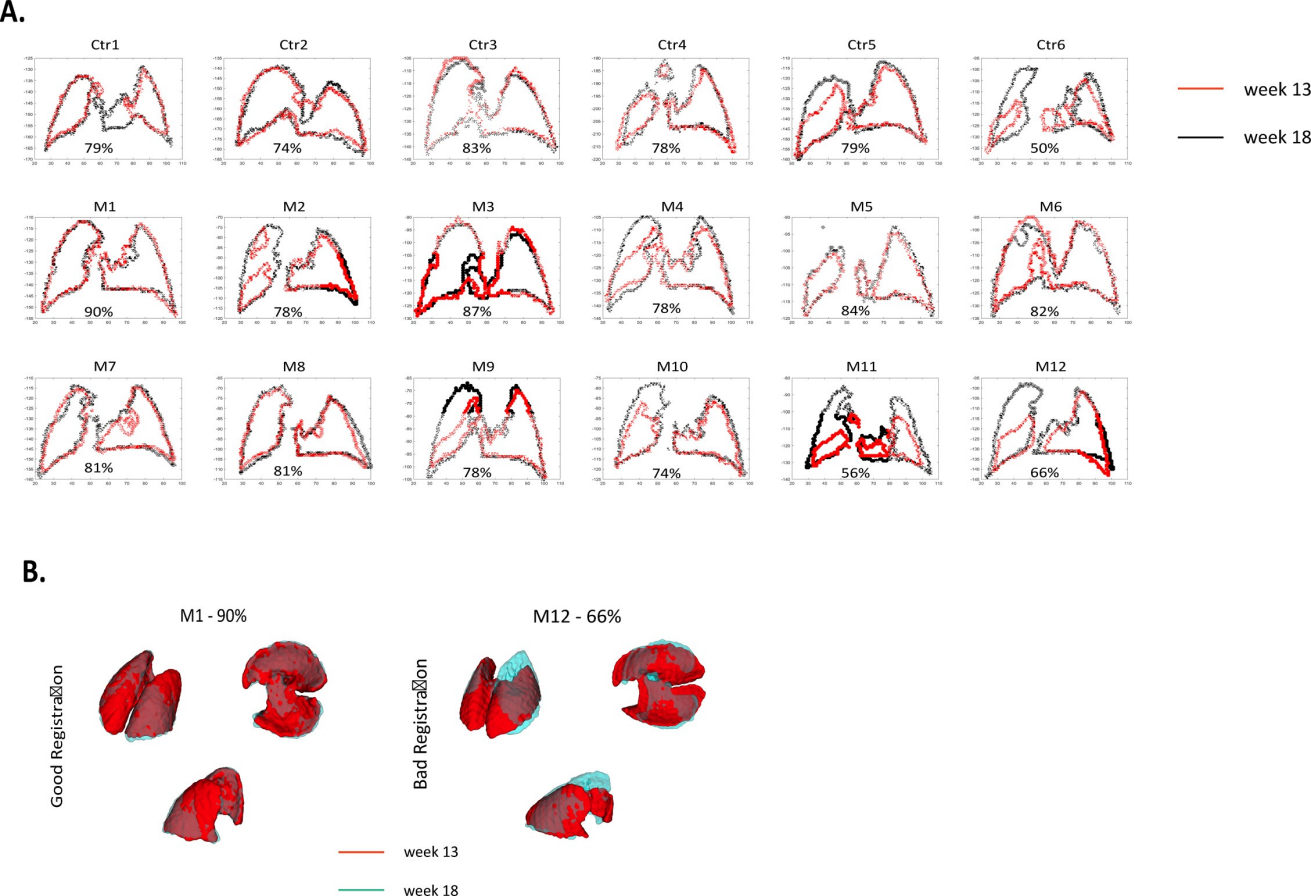
**A)** Cross-section of registered lung mask for week 13 (red) and 18 (black) showing registration quality control check as Dice coefficients. **B)** Example of registered 3D segmentations illustrating good (M1, left-side) and bad (M12, right-side) registrations. Week 13 mask is shown in red and week 18 mask is shown in light blue.

A middle slice of the registered lung masks per mouse can be seen in Fig 3A. A Dice coefficient above 0.800 was considered good and individual tumours in 6/12 mice were easily mapped and quantified semi-automatically through use of a shared seed point for the segmentation. Individual tumour mapping and quantification was also possible for mice with Dice coefficient of 0.778 or above (in three further mice M2, M4, and M9, Fig 3A), but this required manual positioning of the seed points for images acquired at the two time points. For the other three mice, the automatic segmentation technique failed due to atelectasis, but manual volumetry quantification was still possible. As the unambiguous identification of individual tumours from one timepoint to the other was not possible for these mice, no further attempt was made to track the growth of these individually. Further refinements to the analysis technique are in development in order to address this issue.

**Fig 4:**
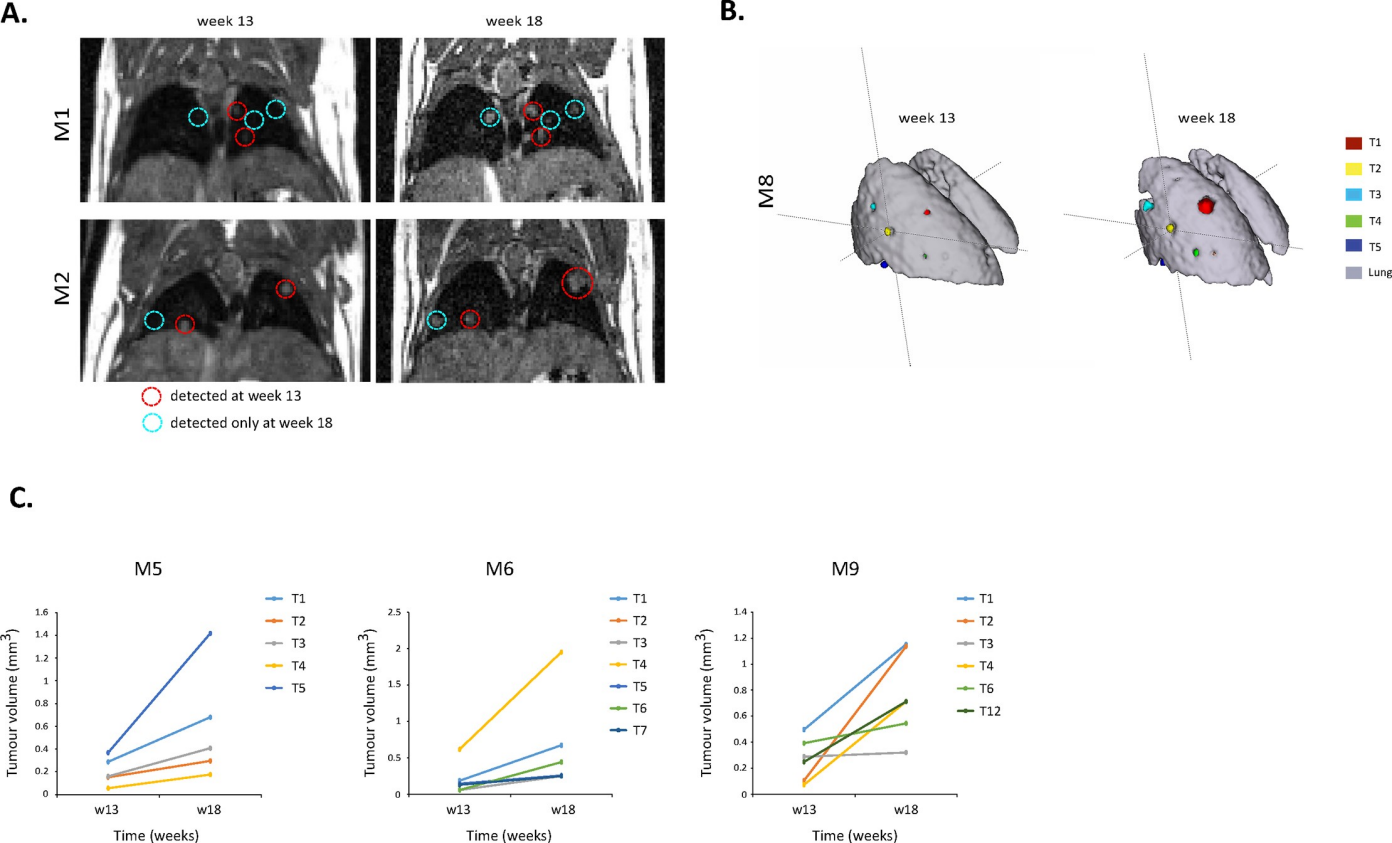
**A)** Coronal images of two mice (M1 and M2), acquired at weeks 13 and 18 post-induction, showing tumour growth. The image acquired at week 13 is registered to the image acquired at week 18. Red circles show tumours present by week 13 (early tumours), while blue circles show tumours present only at week 18 (late tumours). **B)** 3D segmentations of mouse M8 at week 13 and week 18 post induction, showing tracking of individual tumours and their growth. Registered lung masks are shown in grey and individual tumours (T1-T5) are colour coded. **C)** Individual tumour volumes (mm^3^) per time point, in three urethane mice (M5, M6, and M9).

In summary, quantification of individual tumour growth was possible in 9/12 mice, even with tumour volumes estimated to be 0.056 mm^3^ (Table 2). Additional 3D examples of good (M1) and bad (M12) registrations are given in Fig 3B. In all mice, some tumours were detected already at week 13 while other tumours were only visible at week 18 (Fig 4A and 4B). For the tumours already detected at week 13, individual growth rates were different from tumour to tumour, even within the same mouse lung (Fig 4C, Table 2), highlighting the importance of individual tumour quantification and follow-up.

**Table 2:** Volumes (mm^3^) of individual tumours (T1-T18), per mouse (M1-M9), per time point (w13-w18).

	M1		M2		M3		M4		M5		M6		M7		M8		M9	
	w13	w18	w13	w18	w13	w18	w13	w18	w13	w18	w13	w18	w13	w18	w13	w18	w13	w18
**T1**	0.080	0.304	0.136	0.272	0.168	1.072	0.192	0.488	0.288	0.680	0.184	0.672	0.84	1.904	0.936	3.368	0.496	1.152
**T2**	0.072	0.288	0.176	0.248	0.080	1.496	0.104	0.176	0.152	0.296	0.144	0.248	-	0.136	0.176	0.528	0.104	1.136
**T3**	0.160	0.416	0.184	1.184	0.184	0.528	0.056	0.176	0.160	0.408	0.056	0.248	0.200	0.472	0.336	0.592	0.288	0.320
**T4**	0.168	0.752	0.272	0.392	0.128	0.288	0.056	0.120	0.056	0.176	0.616	1.952	0.232	0.608	0.224	0.304	0.072	0.712
**T5**	0.080	0.336	0.192	0.288	-	0.368	-	0.152	0.368	1.416	0.144	0.256	0.144	0.328	0.192	0.488	-	0.296
**T6**	-	0.200	-	0.120	-	0.592	-	0.152	-	0.344	0.056	0.440	0.320	0.552	-	0.072	0.392	0.544
**T7**	-	0.096	-	0.192	-	0.760	-	0.184	-	1.200	0.128	0.248	0.392	0.792	0.184	0.328	-	0.520
**T8**	-	0.152	-	0.152	-	0.480	-	0.200	0.160	0.208	-	0.072	-	0.056	-	0.096	-	0.264
**T9**	-	0.240	-	0.328	-	0.360	-	0.304	-	0.056	-	0.256	-	0.176	-	0.192	-	0.376
**T10**	-	0.480	-	0.256	-	0.416	0.208	0.672			-	0.248	-	0.072	-	0.152	-	0.144
**T11**	-	0.112	-	0.232	-	0.088					-	0.072	0.104	0.232			-	0.080
**T12**	-	0.104	-	0.352	-	0.208					-	0.104					0.248	0.712
**T13**	-	0.184	-	0.304	-	0.608											-	0.344
**T14**	-	0.128		0.104	-	0.072											-	0.056
**T15**	-	0.304		0.200													-	0.152
**T16**	-	0.304		0.472														
**T17**	-	0.320		0.360														
**T18**	-	0.128		0.152														
***TTV***	*0*.*560*	*4*.*848*	*0*.*960*	*5*.*608*	*0*.*560*	*7*.*336*	*0*.*616*	*2*.*624*	*1*.*184*	*4*.*784*	*1*.*328*	*4*.*816*	*2*.*232*	*5*.*328*	*2*.*048*	*6*.*120*	*1*.*600*	*6*.*808*
***AVT***	*0*.*112*	*0*.*269*	*0*.*192*	*0*.*312*	*0*.*140*	*0*.*524*	*0*.*123*	*0*.*262*	*0*.*197*	*0*.*532*	*0*.*190*	*0*.*401*	*0*.*319*	*0*.*484*	*0*.*341*	*0*.*612*	*0*.*267*	*0*.*454*
***SDV***	*0*.*043*	*0*.*160*	*0*.*044*	*0*.*232*	*0*.*040*	*0*.*371*	*0*.*065*	*0*.*170*	*0*.*102*	*0*.*449*	*0*.*179*	*0*.*493*	*0*.*232*	*0*.*503*	*0*.*271*	*0*.*935*	*0*.*149*	*0*.*335*

w13: week13; w18: week 18; TTV: total tumour volume; AVT: average volume per tumour; SDV: standard deviation of population

## Conclusion

We have developed a rapid CR-synchronized MR scan that allows for high throughput in vivo imaging of lung tumour growth, with less than 15 min turnaround time per mouse. When coupled with the described method for semi-automated registration and quantification, individual lung tumours can be followed. This tracking option is the first step to potentially achieve bigger studies in areas such as tumour preferential location and distribution in the lung and in vivo individual tumour growth kinetics in response to different treatments, including chemo and radiotherapy, among others. Such potential for high throughput provides opportunities that include the use of larger sample sizes, more sample groups, more repeated imaging, improved access to limited-availability scanners, and the ability to operate the adaptive trial study designs, optimizing experimental and welfare outcomes.

## Supporting information

S1 Appendix The ARRIVE Guidelines Checklist for this manuscript. (DOCX)
