# Supplementary material for: Cardio-Respiratory synchronized bSSFP MRI for high throughput in vivo lung tumour quantification
Source: PLoS One. 2019 Feb 12;14(2):e0212172. doi: 10.1371/journal.pone.0212172 (PMC6372180; doi:10.1371/journal.pone.0212172)

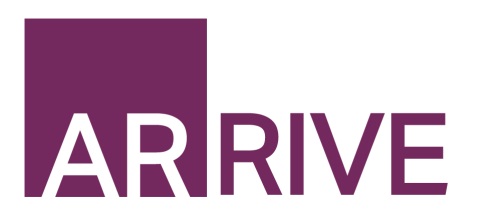


The ARRIVE Guidelines Checklist

Animal Research: Reporting In Vivo Experiments

Carol Kilkenny^1^, William J Browne^2^, Innes C Cuthill^3^, Michael Emerson^4^ and Douglas G Altman^5^

*^1^The National Centre for the Replacement, Refinement and Reduction of Animals in Research, London, UK, ^2^School of Veterinary Science, University of Bristol, Bristol, UK, ^3^School of Biological Sciences, University of Bristol, Bristol, UK, ^4^National Heart and Lung Institute, Imperial College London, UK, ^5^Centre for Statistics in Medicine, University of Oxford, Oxford, UK.*

|  | | ITEM | RECOMMENDATION | Section/ Paragraph |
| --- | --- | --- | --- | --- |
| 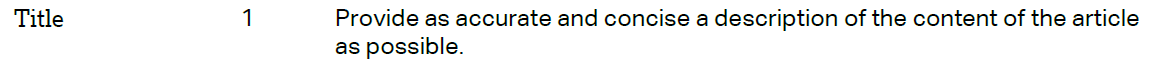 | | | Page 1 |  |
| 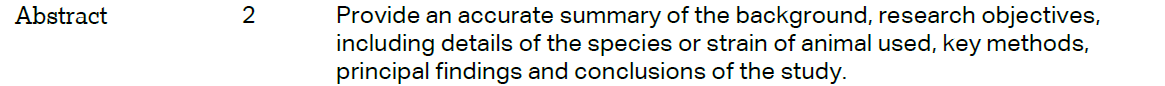 | | | Page 2 |  |
| INTRODUCTION | | |  |  |
| 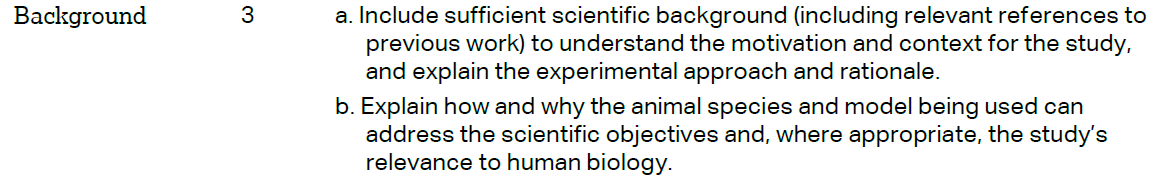 | | | Pages 3-4 |  |
| 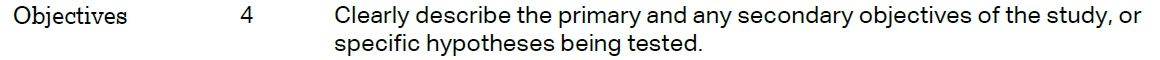 | | | Pages 3-4 |  |
| METHODS | | |  |  |
| 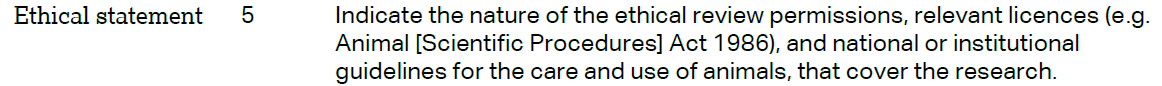 | | | Page 5, first paragraph |  |
| 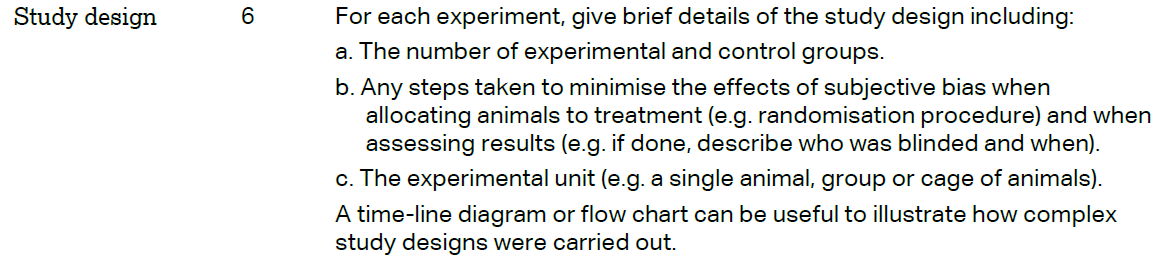 | | | Page 5,  Animal model section |  |
| 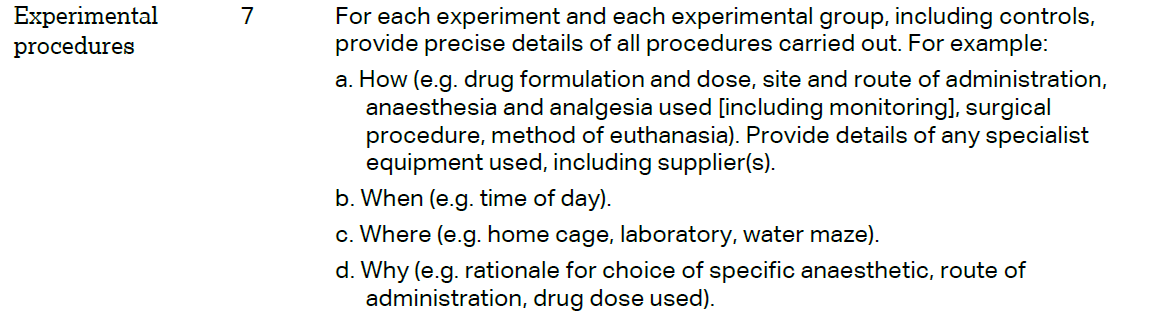 | | | Pages5-6  MR imaging and MR methods  sections |  |
| 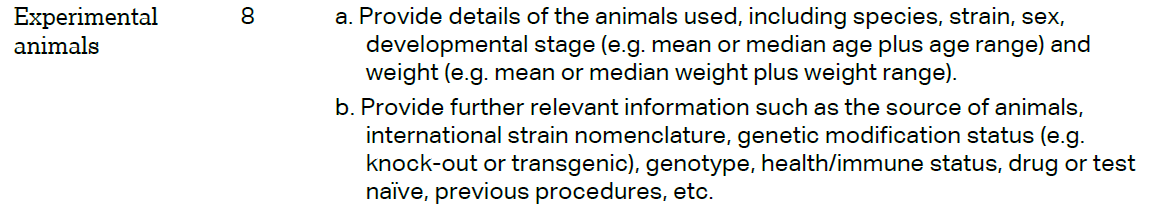 | | | Page 5,  Animal model section |  |

The ARRIVE guidelines. Originally published in *PLoS Biology*, June 2010^1^

| 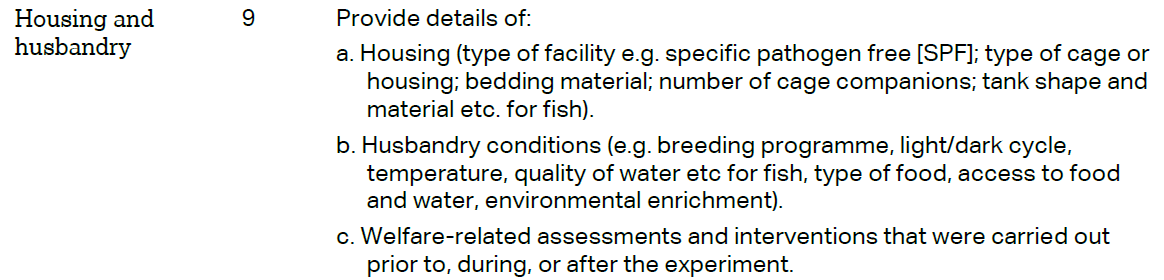 | Page 5,  Animal model section | |
| --- | --- | --- |
| 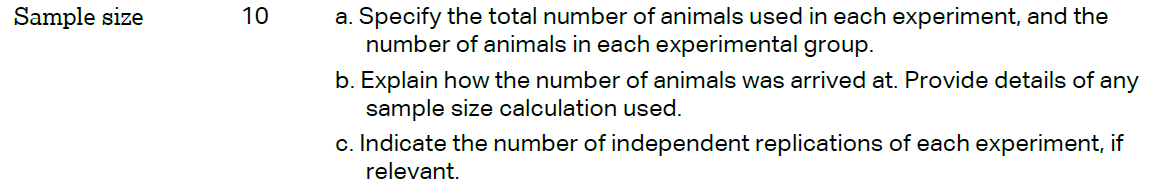 | Page 5,  Animal model section | |
| 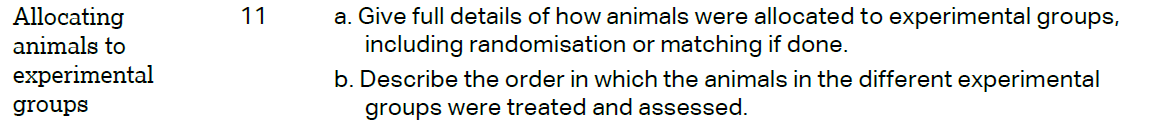 | N/A | |
| 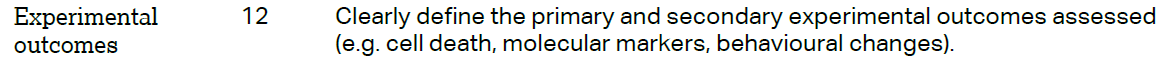 | Page 7, Image Analysis section | |
| 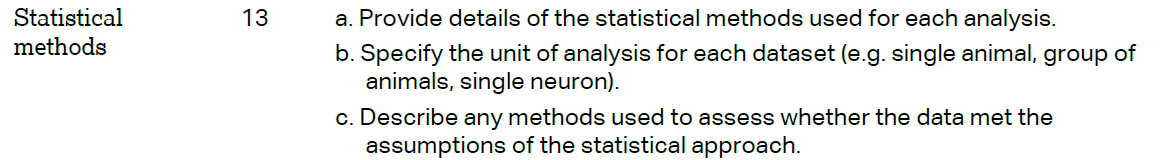 | Page 7,  Statistical Analysis Section | |
| RESULTS |  | |
| 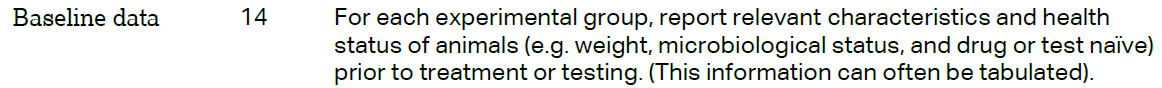 | Weight and drug/naïve group distribution reported on Methods/Animal model section (Page 5), remaining n/a. | |
| 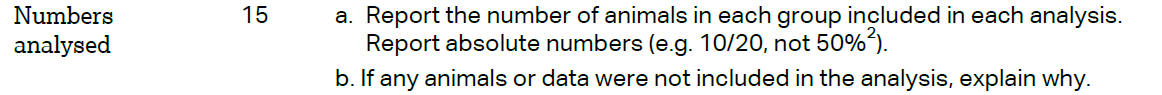 | Figures 2 and 3, and Results section on pages 8-10 | |
| 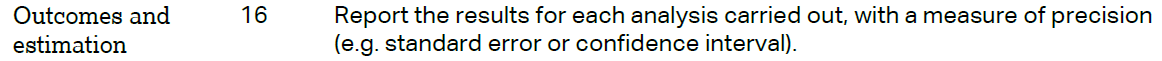 | Tables 1 and 2 | |
| 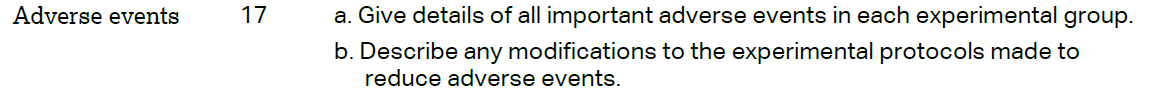 | N/A. no adverse effects were observed. | |
| DISCUSSION |  | |
| 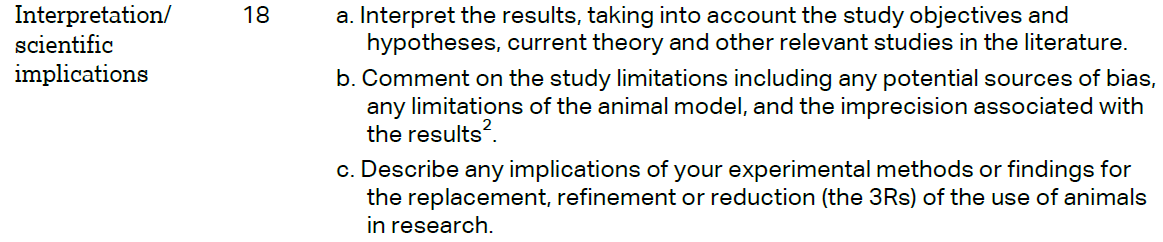 | Pages 8-16 | |
| 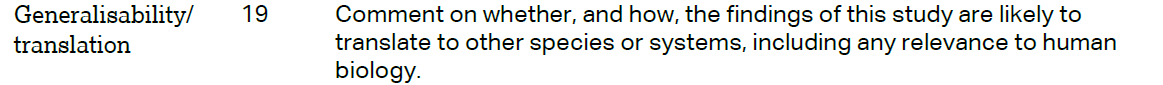 | Page 16, Conclusion section | |
| 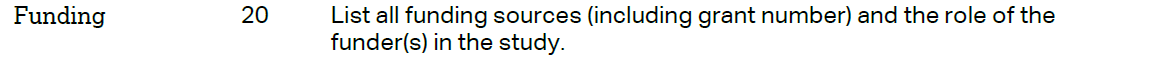 | | Page 16  Funding section |


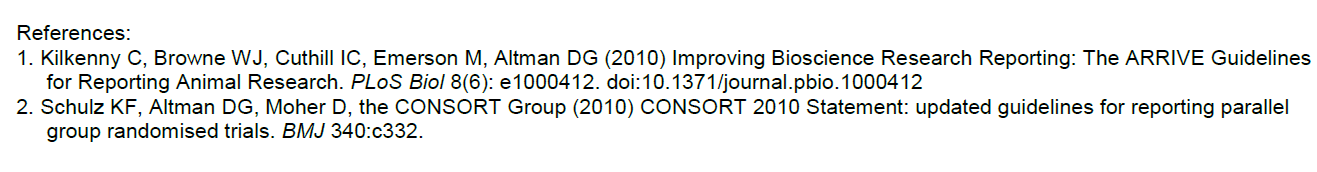

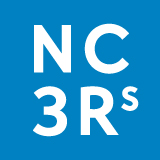

Supplement: S1 Appendix — (DOCX) [file pone.0212172.s001.docx]
